# Does the use of 3D-printed cones give a chance to postpone the use of megaprostheses in patients with large bone defects in the knee joint?

**DOI:** 10.1515/med-2022-0494

**Published:** 2022-07-15

**Authors:** Daniel Kotrych, Sławomir Marcinkowski, Adam Brodecki, Marcin Anuszkiewicz, Jakub Kleszowski, Andrzej Bohatyrewicz, Dawid Ciechanowicz

**Affiliations:** Department of Children Orthopaedics and Musculoskeletal Oncology, Pomeranian Medical University, Szczecin 71-281, >Poland; Department of Orthopedics, Specialist Orthopedic and Rehabilitation Hospital “Gorka,” Busko Zdroj, Poland; Department of Orthopaedics, Traumatology and Orthopaedic Oncology, Pomeranian Medical University, Szczecin, Poland; Department of Orthopaedics, Traumatology and Orthopaedic Oncology, Pomeranian Medical University, Unii Lubelskiej 1, Szczecin 71-281, Poland

**Keywords:** total knee arthroplasty, revision knee arthroplasty, 3D-printed cone augments, metaphyseal bone loss, AORI 3

## Abstract

Revision procedures and the resulting bone loss are a big challenge for orthopedic surgeons. Therefore, we checked what functional outcomes that 3D-printed cone augments can offer to patients with bone defects (Anderson Orthopedic Research Institute [AORI] classification type 2B and 3) in the knee and whether the use of cones can delay the necessity to use a megaprotheses. Data from 64 patients (*M* = 22; *W* = 42) who underwent total knee arthroplasty (TKA) revision were included in the analysis. The Knee Society Clinical Rating System (KSS) and the range of motion in the knee joint were used for the functional assessment. The mean follow-up was 28 months (range: 18–44 months). The survival rate for aseptic loosening at follow-up was 100%. Infection occurred in two (3.1%) patients. The mean KSS score increased from 12.75 points preoperatively to 66.56 postoperatively (*p* < 0.001). The mean range of motion in the knee changed from 61.15° preoperatively to 115.93° postoperatively (*p* < 0.001). 3D-printed cone augments seem to be a good solution for patients requiring a TKA revision procedure. When used in patients with bone defects classified as 2B and 3 (AORI), they can be a good alternative, delaying the need for megaprotheses.

## 1 Introduction

Total knee arthroplasty (TKA) is an effective and commonly used method of treatment for advanced osteoarthritis. All over the world, the number of primary arthroplasty procedures is constantly growing. In 2005, a total of 523,000 joint replacement operations were performed in the United States. It is estimated that this number will increase by 673% to 3,480,000 procedures in 2030 in the United States. The number of revisions in the same year amounted to 38,500 and is expected to increase by over 600% to 268,200 in 2030 in the United States [1,2]. The most common reasons for revision are infections, instability, wrong size and fixation of implants. In the case of late revisions (>2 years from the primary procedure), these are polyethylene wear (34%) and aseptic loosening (24%) [3].

The decision to perform a TKA revision should be considered in many aspects, especially in terms of the expected results. The procedure is associated with a lot of problems. The most important ones are reconstructing bone defects, obtaining a stable and correct component fixation, restoring the joint line, determining the correct traction of the patella and obtaining uniform tension of the collateral ligaments and identical space in the flexion–extension movement [4,5]. The survival of implants after a revision surgery is lower (86% over 10 years) compared to primary surgery (95% over 15 years) [6]. This is due to a more complex clinical situation, mainly related to bone loss and changes in bone density.

The treatment of bone defects has evolved over recent years. Various strategies have been described, mainly depending on the size of the bone defect, including the use of cement, metal augments or bone graft [7]. While the treatment of minor and moderate defects shows good results, treatment methods for major bone defects remain problematic [8], especially in the metaphyseal zone. High hopes can be pinned on the use of cone-type augments, made of porous surfaces, enabling biological integration through bone ingrowth [9]. The aim of this study is to check the clinical results in patients treated surgically with 3D-printed metal cone augments and, in particular, to determine whether the use of metaphyseal augments in patients with type 2 and 3 defects according to Anderson Orthopedic Research Institute (AORI) is a chance to delay the use of resection procedures and megaprotheses.

## 2 Methods

Between January 2017 and January 2020, a total number of 64 knee arthroplasty revisions were performed with the use of the 3D-printed cone augments. All patients or their relatives gave written informed consent to be included in scientific studies during their admission to the hospital. All procedures performed in studies involving human participants were in accordance with the ethical standards of the institutional and/or national research committee and with the 1964 Helsinki declaration and its later amendments or comparable ethical standards and have been approved by the authors’ institutional review board or equivalent committee.

Qualification for surgery was undertaken independently by orthopedic specialists experienced in TKA revision procedures. The decision on the choice of stem length and method of fixation was made by the surgeons based on planning and intraoperative evaluation.

Patients were reviewed retrospectively. The following factors were assessed: the reason for the revision and the number of previous operations; the number of complications (infections, implant loosening and fracture); and integration of the implant with bone tissue (signs of loosening defined as implant migration or radioactivity ≥2 mm along with the entire component). In addition, the functional assessment of the joint, using The Knee Society Clinical Rating System (KSS), was evaluated. The evaluation was conducted pre- and post-operatively in the twelfth month after the surgery.

The dimensions of the components used and the method of fixation were also checked. The level of bone loss was assessed on the basis of preoperative radiological examinations (CT and X-ray) according to the AORI classification, which is one of the most commonly used scales for assessing bone defects. All radiographs were reviewed by five independent orthopedists.

The results of the KSS were statistically analyzed. After checking the data distribution (the Shapiro–Wilk test), Student’s *T*-test was performed in relation to the results in the questionnaire and the range of motion in the knee joint. A *p*-value of <0.05 was considered a statistically significant difference. Statistical analysis was carried out using the Statistica 13.0.2 program.

## 3 Results

### 3.1 Demographic data

The study group (*n* = 64) consisted of 42 women (66%) and 22 men (34%). The mean age of the patients at the time of surgery was 71 years (range: 57–80 years). The mean follow-up was 28 months (range: 18–44 months). In the analyzing group, it was the first revision surgery for 41 (64.1%) patients, 12 (18.8%) patients had two previous operations, 4 (6.2%) patients had three previous operations and 7 (10.9%) patients had four previous operations. In 35 (54.7%) cases, the reason for revision was an incorrect rotation of the endoprosthesis components and pain, loosening in 18 (28.1%) cases, infection in 9 (14.1%) cases and polyethylene wear in 2 (3.1%) cases. Patients who were operated on due to infection underwent a two-stage procedure. The duration between stages was on average 6 weeks (Table 1).

**Table 1:** Demographic data of patients reconstructed with 3D-printed cone

Data	Patients	Percentage
**Age and gender**		
Age (mean years)	71 (range: 57–80)	
Gender (male/female)	22/42	34/66
**Revision surgery**		
First	41	64.1
Second	12	18.8
Third	4	6.2
Fourth	7	10.9
**Reason for revision**		
Incorrect component rotation	35	54.7
Loosening	18	28.1
Infection	9	14.1
Polyethylene wear	2	3.1

### 3.2 Surgical outcomes

For the reconstruction of massive bone defects, 3D-printed cone was used, including 38 (59.4%) tibial parts (13 Central and 25 peripheral) and 26 (40.6%) femoral parts. In 13 (20.3%) cases, cones augmentation involved both the femur and the tibia.

Based on the AORI classification, 65.7% of the tibial defects (*n* = 25) and 7.7% of the femoral defects (*n* = 2) were classified as type 2B using pre-operative radiographs, computer tomography and intraoperative evaluation, while 34.2% of the tibial bone loss (*n* = 13) and 92.3% of the femoral bone loss (*n* = 24) were classified as type 3.

On the tibia side, 64 stems were used, including eight (12.5%) fixed with cement. On the femoral side, 64 stems were used, all uncemented. The length of tibial stems was 100 mm in 49 (76.6%) knees, 50 mm in 8 (12.5%) knees and 150 mm in 7 (10.9%) knees. On the femoral side, all stems were 100 mm long. In one case, a patella prosthesis was implanted. Radiographic analysis showed bone–cone integration in all cases at 98% of the border bone area (Table 2).

**Table 2:** Summary of operational outcomes in the study group, treated with 3D-printed cones (*n* = 64)

Data	Patients	Percentage
**3D-printed cone**		
Tibial part	38	59.4
Femoral part	26	40.6
Both	13	20.3
**Bone defects (AORI)**		
Type 2B	27	42.2
Type 3	37	57.8
**Type of fixation**		
Tibia (cem.^1^/uncem.^2^)	8/56	12.5/87.5
Femur (cem.^1^/uncem.^2^)	0/64	0/100
**Length of the tibial stems**		
50 mm	8	12.5
100 mm	49	76.6
150 mm	7	10.9
**Length of the femoral stems**		
100 mm	64	100

^1^cem. – cemented.

^2^uncem. – uncemented.

### 3.3 Complications

The survival rate for aseptic loosening at follow-up (28 months) was 100% (95% CI = 95–100%). Deep infection (*S. aureus*) occurred in two (3.1%) patients 6 weeks after implantation. In our group, a total of 14 (21.9%) patients underwent additional operations. The most common treatments are irrigation and debridement. No intraoperative complications related to cone implantation were reported.

### 3.4 Functional results

**Figure 1:**
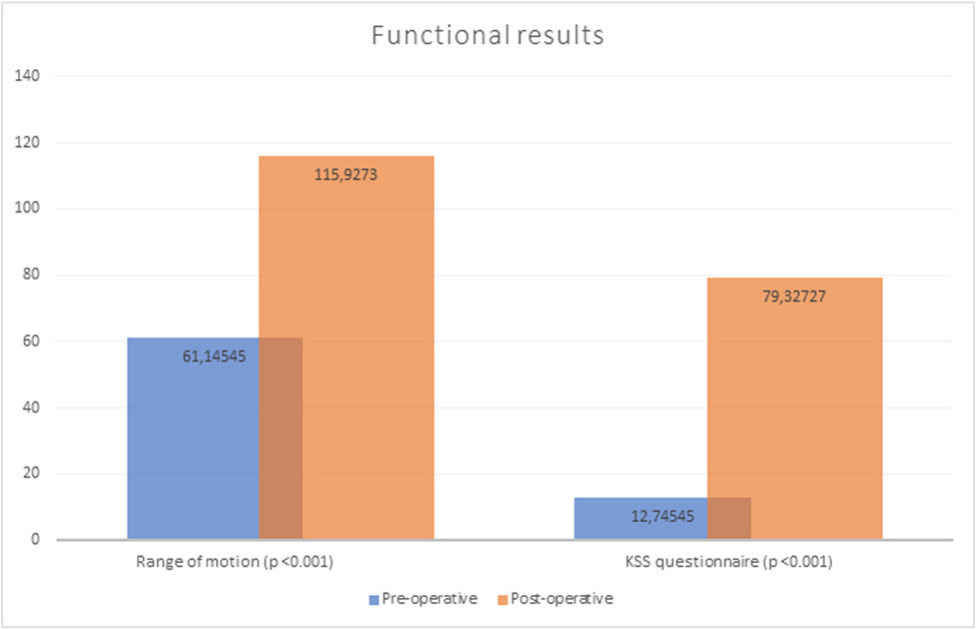
Comparison of patients’ functional outcomes (KSS questionnaire and range of motion) in the preoperative and 12 months postoperative period.

The mean preoperative range of motion in the knee was 61.14545° (±6.059636°). After surgery, it increased by an average of 54.78 points (*p* < 0.001) to 115.9273° (±6.624462°). The opening mean score in the 100° KSS questionnaire was 12.74545 (±7.506092) points, after the procedure increased by an average of 66.56 points (*p* < 0.001) to 79.32727 (±3.288896) points (Figure 1).

## 4 Discussion

Revision procedures after TKA are often a big challenge for surgeons. Additional problems arise especially when large bone defects occur (2B and 3 according to the AORI classification). According to our experience and the available literature, the key to the success of the procedure is appropriate planning, which takes into account, e.g., zonal classification system. It determines three levels of implant fixation: joint surface, metaphysis and diaphysis [10]. Most revision systems achieve stability due to the distribution of loads on the joint surface and diaphysis zones; however, it is in the metaphyseal zone that the greatest forces occur, where their uneven distribution leads to loosening [11]. Understanding the role of fixation zones allows us to create a methodology of conduct. The components should be firmly seated with a proper distribution of mechanical forces, which is a decisive success factor. Lack of uniform load leads to movements exceeding 150 μm, which stimulates the formation of soft tissue, preventing implant osteointegration [12]. Methods of treating bone defects have been described in detail in the literature, especially during the short- and medium-term follow-up period. Smaller, closed defects can be treated with a bone substitute or bone cement, whereas the larger ones can be treated with bolt-reinforced cement or modular metal parts. The joint surface defects require the use of structural grafts or metal implants [5]. The evolution of materials has contributed to the development of porous surfaces that improve mechanical stability. To provide a structural and biomechanical reconstruction of the metaphyseal area, titanium implants can be used to increase the surface area and support the remaining implants. These solutions include sleeve’s and cone’s, which are two different technologies with different philosophies. In our practice, we decided to use cones for several reasons. One of them is the independence of zone implantation, which enables the distribution of forces beyond the articular area, protecting it from excessive stresses. The fixation in the metaphyseal zone is independent of the joint surface and diaphysis zones. Ensuring the independence of the positioning of the tibial tray and the extension allows for >85% of the adjustment of the stem to the bone shaft without disturbing the mechanical axis [13]. In other cases, the fit can be achieved using a cemented stem. Another reason is the covering of the implant modeled on the structure of the trabecular bone. An average porosity of 80% combined with a modulus of elasticity, low stiffness and a high coefficient of friction increases osteoconductive, ensuring physiological load transfer and reducing the risk of loosening [14]. A great advantage of cones is the low potential for bacterial adhesion and high survival [15]. Thanks to the use of titanium alloy (Ti_6_Al_4_V), cones are characterized by high corrosion resistance, excellent biocompatibility and very good osteointegration. In addition, Allizond et al. present the ability of a Ti_6_Al_4_V surface nanotexture to limit bacterial adhesion, compared to a mirror-polished control, even without the addition of silver [16]. All these features translate into the longevity of the implants. Bonanzinga et al. conducted a meta-analysis of 432 implanted cones with an average follow-up of 42 months. Features of loosening were shown by 1.15% [17].

**Figure 2:**
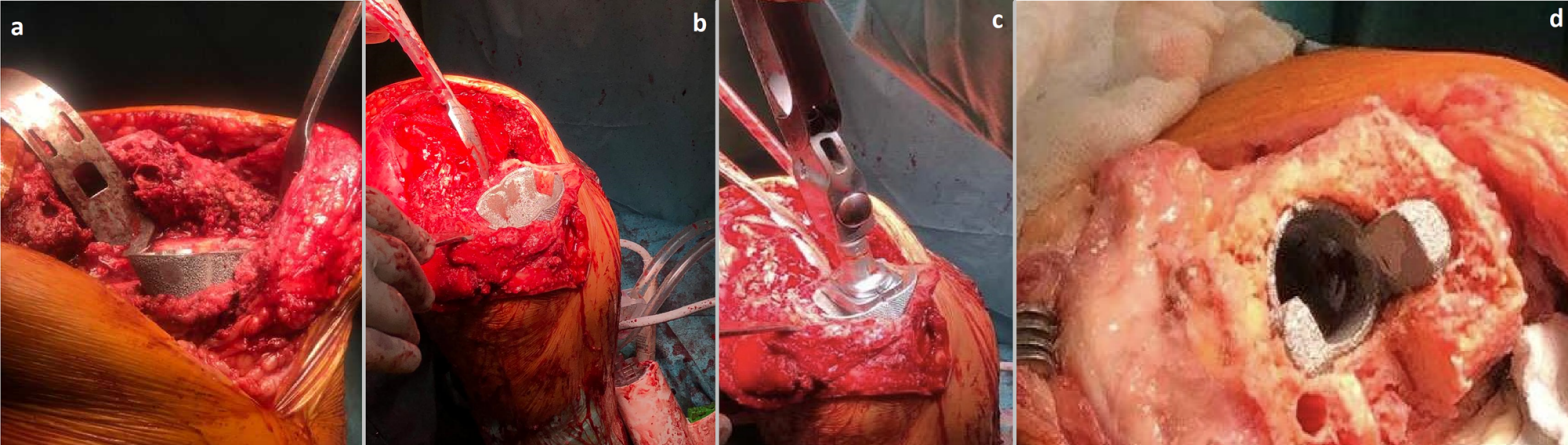
Intraoperative view of 3D printed Cone auguments; (a) Total reconstruction of the condyle in the metaphyseal zone of the tibia. The outer layer of the implant provides high porosity, which translates into a friction increase in osteoconductive. (b and c) The use of the peripheral Cone to rebuild the tibial surface resigning from the use of a tibial augment; (d) The example of femoral Cone where broken bone fragments with ligament attachments were based on the implant.

The change in bone density (on average by 27%) after primary arthroplasty was correlated with its severe loss, and it is a serious challenge in TKA revision [14]. 3D-printed titanium cones with anatomical shapes and porous structure can provide structural support while offering the potential for permanent biological fixation. The use of structural grafts has been associated with long remodeling times, limited graft availability, nonunion or resorption. Admittedly, Sandiford et al. proved comparable effectiveness in the application of both solutions; however, most reports indicate a high revision rate exceeding even 20% (over a 10-year period) [18]. The first generation of cones showed promising clinical and radiological results in TKA revisions. Their limitations were imprecise bone preparation and limited availability of sizes and shapes. The second generation offers a much wider range and safer preparation [19].

The production technique based on 3D printing allowed the creation of a wide range of sizes for high fit, based on the use of a large CT database to determine the location and shape for optimal bone coverage and support [20]. The tools adapted to the size significantly reduced the risk of uncontrolled milling without obtaining a guarantee of geometric fit and the need to use cement. Faizan et al. compared traditional and 3D-printed implants. In the case of using traditional implants, the incidence of intraoperative fractures during preparation was 4%. By analyzing movements during valgus and simulated steps, the research showed similar values for the use of central tibial cones for both technologies. In the case of asymmetrical tibial and femoral cones, they were much smaller compared to traditional [21]. The benefits of using an implant based on 3D printing were described by Patel et al., assessing the stability and radiological results from the third to the sixth month after surgery as 100% [22]. Denehy et al. performed a multicenter review of 62 TKA revisions using 3D cones, with at least 2 years of follow-up. There were no cases of aseptic loosening and the survival rate was 90.2% [20]. In both studies, the reason for the review was infections. The authors noted no signs of loosening in radiological examinations or progressive lines translucent to X-rays (after excluding patients with infection).

In this study, the mean score in the KSS questionnaire improved from 12.5 to 79.3 points. Similar results were reported by Girerd et al. [23]. Tetreault et al. also showed 98% survival over a 2-year period. The authors observed four cases of unsuccessful osseointegration [24]. Divano et al. reported 100% aseptic survival in the mean 5 years and 2% revision due to infection, treated without the need for Cone removal. At the same time, the authors indicated 96% survival of the first-generation femoral Cone in a series of 159 cases but with a 24% fracture rate. The complication rate for 3D cones was 2.1% [25]. A potential failure may be the use of cones in sclerotic bone, which is a serious obstacle to osteointegration, especially on the tibia side where the risk of loosening is greatest [14,19,24]. You et al. presented no deep infection in 17 cases with a mean follow-up of 3.5 years [26]. Our study also reported a very low rate of infections – two cases (1.2%) within the first 6 weeks after surgery.

So far, severe bone defects, classified as type 3 according to AORI, have mostly been treated with a structural graft or resection prostheses [27]. By using the multi-zone fixation technique, we believe that cones can also be effectively used in such cases. Their use allows us to raise the level of implant fixation, ensuring the correct reconstruction of the joint line, while avoiding further bone resection and sharing the load forces between the metaphyseal and join surface zones. Such an application is described by Kukreja and Swanson presenting a series of six cases (AORI type 3) using the tibial cones. Radiographic evaluation showed 100% of osteointegration recorded on final radiographs in all patients after a mean follow-up of 4.1 years [28]. Similarly, Meneghini et al. described the use of Cone implants in the reconstruction of the tibia and joint line as an effective solution for delaying the use of a respectable endoprosthesis [29]. Our experience also shows this possibility. In nine cases, we reconstructed the metaphyseal zone of the tibia using the peripheral Cone with total reconstruction of the condyle (Figure 2a). In four cases, we used the peripheral Cone to rebuild the tibial surface, resigning from the use of a tibial augment (Figure 2b and c). This decision was dictated by osteoporotic bone tissue and the belief in a more favorable, superficial force distribution into the diaphysis zone. In three patients, the broken bone fragments with ligament attachments were based on the femoral Cone and, in one case, reinforced with titan wire loops (Figure 2d). The radiological evaluation of the image over a 2-year period did not reveal any signs of loosening. Physical examination confirmed ligamentous stability with a range of motion of 0–120. In one case, we used the femoral and tibial cones to fill the defects after removing the loosened sleeves, and in another case, after removing the hinged prosthesis, which effectively allowed us to fill the defects classified as AORI 3 type. We also believe that the use of cones allows us to reduce the need to use resection prosthesis in doubtful cases. Their use allows us to rebuild the destroyed metaphyseal zone and restore the joint line. However, in the case of significant bone loss, reaching diaphysis, we recommend the use of resection prostheses with fixation in the bone shaft.

This study has limitations related to a retrospective analysis carried out on a heterogeneous group of patients. However, the effects of the retrospective nature of the study are mitigated by the prospective collection of data. It is worth adding that this is one of the few studies of a series of cases with the use of 3D cones in a large number of patients undergoing functional assessment. The use of implants was determined by surgeons with no preferred indication of a particular type. Another limitation is the imperfection of the KSS scale as a measure of clinical outcome that combines subjective outcomes with implant survival data. However, it is worth adding that it is the most frequently used classification for functional assessment of patients after TKA. Finally, the length of the observation is relatively short. However, 3D-printing Cone implants were introduced in Poland in 2017. Therefore, further monitoring of our population will provide additional information.

## 5 Conclusion

The use of cone implants has gained importance in the revision of the knee joint due to its biological and mechanical properties. Structural stiffness and stability due to the high coefficient of friction provide a structural reinforcement in the weaker spongy bone of the metaphyseal zone, ensuring the correct distribution of mechanical forces. Their use could reduce the risk of infection. The available literature and our experience confirm the assumption that cones is a real option in the effective treatment of bone loss classified according to AORI as type 2B and 3. Summarizing the available data, we conclude that 3D-printed cones provide good strengthening of the metaphyseal zone in the short term. These results are encouraging, although they must be confirmed by longer observation.
